# Dissolution of Ag Precipitates in the Cu–8wt.%Ag Alloy Deformed by High Pressure Torsion

**DOI:** 10.3390/ma12030447

**Published:** 2019-02-01

**Authors:** Anna Korneva, Boris Straumal, Askar Kilmametov, Robert Chulist, Grzegorz Cios, Brigitte Baretzky, Paweł Zięba

**Affiliations:** 1 Institute of Metallurgy and Materials Science, Polish Academy of Sciences, 25 Reymonta Street, 30-059 Krakow, Poland; r.chulist@imim.pl (R.C.); p.zieba@imim.pl (P.Z.); 2 Institute of Solid State Physics and Scientific Center of RAS, Russian Academy of Sciences, Ac. OssipyanStr. 2, 142432 Chernogolovka, Russia; straumal@mf.mpg.de (B.S.); askar.kilmametov@kit.edu (A.K.); 3 Karlsruhe Institute of Technology (KIT), Institute of Nanotechnology, Hermann-von-Helmholtz-Platz 1, 76344 Eggenstein-Leopoldshafen, Germany; brigitte.baretzky@kit.edu; 4 National University of Science and Technology «MISIS», Leniskijprosp. 4, 119049 Moscow, Russia; 5 AGH University of Science and Technology, Academic Centre for Materials and Nanotechnology, 30 Mickiewicza Av, 30-059 Krakow, Poland; grzegorz.cios@agh.edu.pl

**Keywords:** Cu–Ag alloy, high-pressure torsion, ultrafine microstructure, phase dissolution, microhardness

## Abstract

The aim of this work was to study the influence of severe plastic deformation (SPD) on the dissolution of silver particles in Cu–8wt.%Ag alloys. In order to obtain different morphologies of silver particles, samples were annealed at 400, 500 and 600 °C. Subsequently, the material was subjected to high pressure torsion (HPT) at room temperature. By means of scanning and transmission electron microscopy, as well as X-ray diffraction techniques, it was found that during SPD, the dissolution of second phase was strongly affected by the morphology and volume fraction of the precipitates in the initial state. Small, heterogeneous precipitates of irregular shape dissolved more easily than those of large size, round-shaped and uniform composition. It was also found that HPT led to the increase of solubility limit of silver in the copper matrix as the result of dissolution of the second phase. This unusual phase transition is discussed with respect to diffusion activation energy and mixing enthalpy of the alloying elements.

## 1. Introduction

Over the last decade, Cu–Ag alloys have attracted attention due to their high strength and high conductivity [1,2,3]. The mechanical and electrical properties of these alloys with low Ag content mainly depend on the volume fraction and distribution of Ag precipitates. Small Ag precipitates act as obstacles against the dislocation movement, increasing both strength and conductivity when Ag is extracted from the Cu matrix. It was found that applying cold drawing to the Cu–6wt.%Ag alloy resulted in a significant increase of strengthening effect from Ag precipitates (from 100 to 560 MPa) at the drawing strain equal to 6 [3]. In this process, fine Ag precipitates are produced which are elongated and evolve into filamentary structure along the drawing direction. It can be assumed that applying severe plastic deformation (SPD) with higher strain should further increase the strength of the material conserving its high conductivity. 

It is well known that SPD allows producing bulk ultra-fine-grained materials with extraordinary mechanical properties, such as exceptionally high strength with considerable ductility at room temperatures or large super-plasticity at elevated temperatures [4,5,6]. Recently, it has been shown that the presence of the second phase (precipitates) additionally influences the grain refinement when the material is subjected to SPD [7,8]. This effect depends on morphology of the second phase (size, volume fraction, distribution). For instance, it has been shown in [8] that an extensive grain refinement of the Al–5.4%Mg–0.5%Mn–0.1%Zr alloy under equal-channel angular pressing (ECAP) was facilitated by a dispersion of Al_6_Mn particles with an average size of 25 nm. They precipitated during the homogenization annealing at intermediate temperature. On the contrary, the formation of coarse recrystallized grains took place in this material after ECAP, when coarse Al_6_Mn particles with a plate-like shape were present in the initial state. On the other hand, the second phase is also affected by SPD, because SPD frequently induces phase transformations [9,10,11]. For example, some phases precipitate or dissolve after SPD at room temperature [12,13,14], which usually should happen after heat treatment at higher temperatures. W. Huang [15] reported that the dissolution of the second phase occurring in SPD is an important phenomenon of phase transition. The dissolution of the second phase induced by SPD also depends on distribution and morphology of the particles. The influence of dissolved precipitated phases on the grain refinement and improvement of mechanical properties of severely deformed materials was reported in [16,17], however there is no systematic research about the effect of SPD on the behavior of particles, with respect to their morphology and properties. Therefore, the aim of this work is to study the influence of high-pressure torsion (HPT) on dissolution of the second phase and microhardness of the Cu–8wt.%Ag alloy characterized by different morphology of Ag precipitates. 

## 2. Materials and Methods

The Cu–8wt.%Ag alloy was manufactured by induction melting in vacuum from the high-purity 5N5 Cu and 4N5 Ag. The resulting ingots with the diameter of 10 mm were cut by means of spark erosion into 0.7 mm thick disks. Three disks of the Cu–8wt.%Ag alloy were sealed separately into quartz ampoules (HMB Quarzglass, Helmenhorst, Germany) with the residual pressure of 4 × 10^−4^ Pa for annealing in a SUOL resistance furnace (Tula-Term, Tula, Russia) at 400 °C (1200 h), 500 °C (710 h) and 600 °C (86 h). After annealing, the samples were quenched in water (ampoules were broken). Afterwards, discs were subjected to HPT in a Bridgman anvil chamber (W. Klement GmbH, Lang, Austria) at a pressure of 5 GPa, five revolutions at the speed of 1 rpm at room temperature. In our experiment three discs for each state were deformed and then examined with X-ray diffraction (XRD, Siemens, München, Germany) technique. Since the obtained XRD-curves were identical for each state, one deformed disc was used for further microstructural investigations using scanning electron microscopy (SEM, FEI, Hillsboro, OR, USA) and transmission electron microscopy (TEM, TECNAI, Hillsboro, OR, USA) techniques. The samples for the structure studies were cut at the distance of 3 mm from the center of the deformed discs. For the metallographic investigations, the samples were ground with SiC grinding paper, and sequentially polished with 6, 3 and 1 μm diamond pastes. The detailed microstructure observation and electron backscatter diffraction (EBSD) analysis were carried out on a FEI Quanta 3D FEGSEM scanning electron microscope (SEM, FEI, Hillsboro, OR, USA) equipped with a field emission gun (FEG) and an energy-dispersive X-ray spectrometer (EDX) manufactured by EDAX (Mahwah, NJ, USA). The SEM images were taken using backscattered electron signal (BSE mode) in order to obtain the composition contrast between different phases. The EBSD maps combined with element maps were carried out on a sample plane perpendicular to the normal direction. The working voltage and current were set to 20 KV and 8 nA, respectively. The mappings were carried out in the beam-scanning mode with a step size of 50 nm. Crystallographic and microstructural features were analyzed with the TSL OIM software (version Analysis 7 EDAX, Mahwah, NJ, USA). In order to visualize orientation maps the so-called inverse pole figure (IPF) color coding was used. The details of the resulting microstructure components, especially in nanoscale, were revealed using a TECNAI G2 FEG super TWIN (200 kV) transmission electron microscope (TEM, TECNAI, Hillsboro, OR, USA) equipped with an energy dispersive X-ray (EDS) spectrometer manufactured by EDAX TECNAI (Mahwah, NJ; USA). Thin foils of the alloy for TEM observation were prepared by a twin-jet polishing technique using D2 electrolyte of Struers company (Ballerup, Denmark) with parameters recommended by the manufacturer. The X-ray diffraction patterns were obtained in the Bragg–Brentano geometry on a Philips X’Pert powder diffractometer with the use of Cu-*K*α radiation. Lattice parameters were evaluated by the Fityk software [18] using a Rietveld-like whole profile refinement. Relative amounts of Cu- and Ag-rich solid solutions were estimated from the integrated intensities. Pure polycrystalline copper was used as reference. The phases in the alloys were identified by comparing with the X’PertHighScorePANalytical phase database [19]. The hardness measurements were performed using an G200 nanoindenter (KLA-Tencor, Milpitas, CA, USA) with XP head with the indentation load of 1.96 mN at the distance of 3 mm from the center of the deformed disc. There were 100 microhardness measurements taken on each sample with 10 × 10 points with the measurement step of 3 µm. After that, each measurement was categorized as copper matrix or silver particle by means of SEM observations. The indents lying on the boundaries between matrix or particles were discarded. A minimum of ten measurements for both phases were taken into account. Low force was chosen to measure hardness of particles and matrix because the particles were small. The same force for both particles and matrix were used in order to compare them. The size of the indenter imprint was about 0.5 and 0.26 µm before and after HPT, respectively.

## 3. Results and Discussion

**Figure 1:**
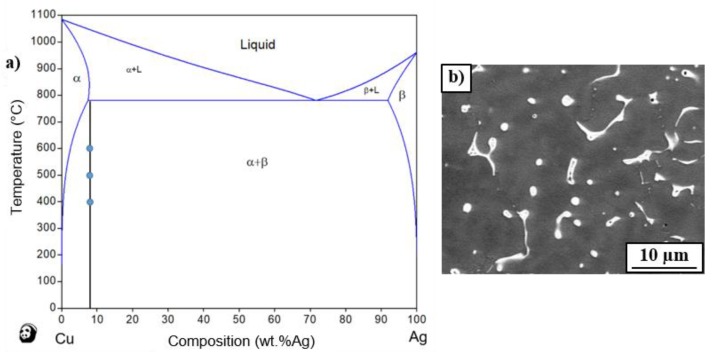
(**a**) phase diagram of Cu–Ag system [20] with vertical line showing chemical composition of the examined alloys, while dots show annealing temperatures of examined samples; (**b**) SEM image of the as-cast Cu–8wt.%Ag alloy.

The Cu–Ag equilibrium phase diagram is presented in Figure 1a [20]. The vertical line shows the chemical composition of the examined alloy, while the dots show the annealing temperatures, i.e., 400, 500 and 600 °C. These temperatures correspond to the (α + β) state, where the α-phase is the solid solution of Ag in Cu whereas the β-phase is the solid solution of Cu in Ag. 

**Figure 2:**
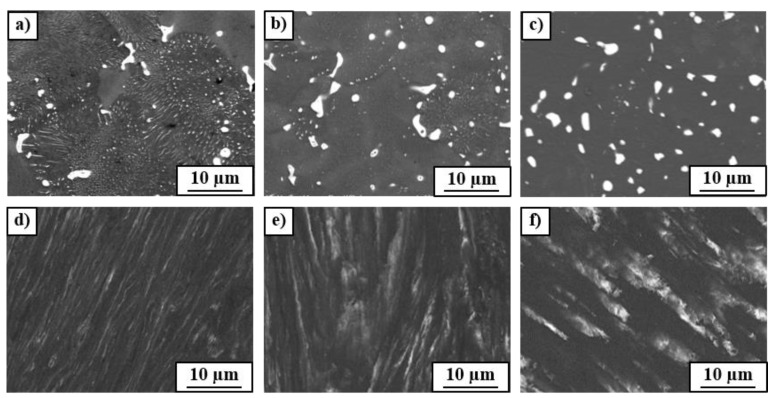
SEM images of the Cu–8wt.%Ag alloy after annealing at (**a**) 400 °C; (**b**) 500 °C; (**c**) 600 °C and after HPT deformation: (**d**) 400 °C + HPT; (**e**) 500 °C + HPT; (**f**) 600 °C + HPT.

**Figure 3:**
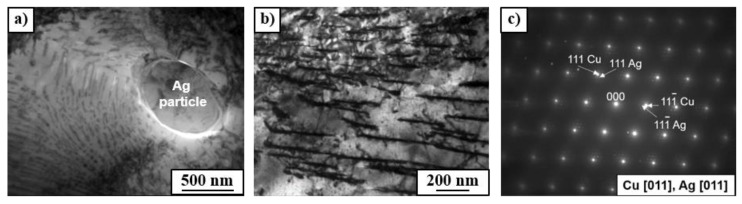
(**a**,**b**) Bright field TEM images of the Cu–8wt.%Ag alloy after annealing at 400 °C and (**c**) SAED pattern taken from image (**b**).

The SEM microstructure observations show that the as-cast Cu–8wt.%Ag alloy contains the precipitates of (α + β) eutectic with irregular shape and small rounded particles of the β-phase of about 1 µm size) uniformly distributed in the α-phase (Cu-matrix), Figure 1b. The eutectic and β particles were enriched with silver (from 11 to 55 wt.%). The chemical composition of as-cast Cu-matrix was inhomogeneous: the Ag content changed from 3.6 ± 0.5 to 6.7 ± 0.3 wt.%. Annealing the as-cast alloy at 400 °C resulted in the preservation of (α + β) eutectic and β-phase particles with the average size of about 1.1 µm. The β-phase particles were observed within the grains and at the grain boundaries of the Cu-matrix (Figure 2a). Annealing at 400 °C resulted also in the discontinuous precipitation (DP) of a duplex structure containing a new fine β-phase and the solute depleted initial α-phase (see SEM image in Figure 2a and TEM images in Figure 3a,b). The DP phenomenon was frequently observed in Cu-based alloys [21,22] including Cu–Ag alloys with low Ag content [3]. A similar microstructure of DP was observed in the as-cast Cu–6wt.%Ag alloy after ageing at 450 °C for 32 h [3]. The DP process was controlled by the diffusion at the moving reaction front between the supersaturated Cu-matrix and forming (α + β) lamellae. The moving reaction front corresponded to the high-angle grain boundary. The selected area electron diffraction (SAED) pattern (Figure 3c) of the bright field image presented in Figure 3b confirms that the observed duplex structure is related to fine β precipitates with the thickness of about 50 nm in the depleted initial Cu-matrix. The measurement of chemical composition by means of EDS in TEM showed that the silver content in the primary β particles remained after casting, (see Figure 3a) was about 87 wt.%Ag, in the fine β precipitates around 30 wt.%Ag, while in the Cu-matrix it was 0.9 wt.%Ag.

The microstructure of the Cu–8wt.%Ag alloy after annealing at 500 °C (Figure 2b) also contained the eutectic, primary β-phase particles and fine β-phase lamellae from the discontinuous precipitation, however the DP volume fraction is much lower than that in the sample annealed at 400 °C. The microstructure of the Cu–8wt.%Ag alloy after annealing at 600 °C differs significantly from that described above; only one type of homogeneous β-phase particles with the average sizes of about 1.4 µm were observed (Figure 2c). It is well known that discontinuous precipitation basically occurs at low temperatures when grain boundary diffusion dominates over bulk diffusion. Generally, the increase of temperature causes the increase of the bulk diffusion coefficient in comparison with the grain boundary one. This is because the activation enthalpy of bulk diffusion is nearly two times higher than that of grain boundary diffusion. Therefore, the increase of annealing temperature resulted in the reduction of discontinuous precipitation at 500 °C and its complete disappearance at 600 °C. The increase of bulk diffusion taking place through the volume of crystal grains at 600 °C explains also the transformation of (α + β) eutectic precipitates into the homogeneous β-phase particles. The measurement of volume fraction of the β-phase on the basis of X-ray diffraction (XRD) patterns showed that with increasing annealing temperature, the volume fraction of the β-phase decreases from about 8.6 to 6.3% (Table 1). This trend correlates with the lever rule for the calculation of the relative number of phases in a two-phase mixture in a binary alloy system. It should be noted that the amount of the β-phase measured in XRD patterns includes all β precipitates in the material; namely, primary ones, those from the eutectic and from the discontinuous precipitation. It should be also noted that according to the equilibrium Cu–Ag phase diagram, the higher the temperature of annealing, the higher the solubility of Ag in the Cu-matrix. The measurement of chemical composition by means of EDS/SEM confirmed that with increasing annealing temperature, the silver concentration in the Cu-matrix (α-phase) and in the coarse β-phase particles grew (Table 2). 

**Figure 4:**
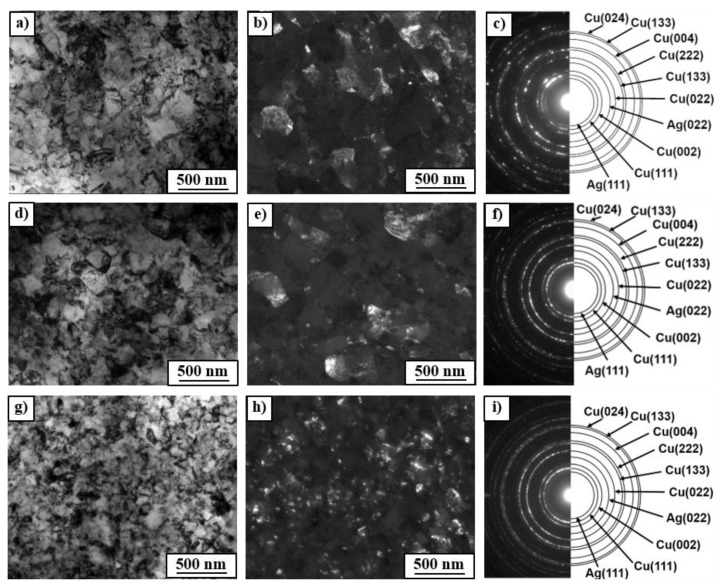
TEM images of the Cu–8wt.%Ag alloy after annealing and HPT: (**a**–**c**) 400 °C + HPT; (**d**–**f**) 500 °C + HPT; (**g**–**i**) 600 °C + HPT; (**a**,**d**,**g**) bright field images; (**b**,**e**,**h**) dark field images; (**c**,**f**,**i**) SAED patterns taken from (**b**,**e**,**h**).

The observation of microstructure of the Cu–8wt.%Ag alloy after HPT by means of TEM technique showed that HPT resulted in a strong grain refinement of Cu-matrix (down to about 400 nm) with high density of dislocations within the grains (see bright and dark field TEM images in Figure 4). The selected area electron diffraction (SAED) patterns (Figure 4c,f,i) also showed a strong grain refinement; a large number of spots formed almost continuous diffraction rings which indicates the occurrence of large quantity of small grains with different crystallographic orientation. The maximum number of spots in the diffraction rings were observed in the deformed sample after prior annealing at 600 °C. It seems that the higher temperature of preliminary annealing, the stronger grain refinement of Cu-matrix. However, the EBSD measurements and XRD diffraction analysis with much better statistics showed the reverse dependencies of grain refinement on temperature of preliminary annealing (see this in the subsequent part of manuscript). A close examination of diffraction rings (Figure 4c,f,i) also showed the presence of small Ag particles in all deformed samples. Most likely, they are related to the fragmentation and refinement of β-phase precipitates (enriched by Ag) due to the HPT deformation. The analysis of their behavior under HPT was additionally carried out using SEM. The SEM observations of deformed samples of the Cu–8wt.%Ag alloy showed blurring of the (α + β) eutectic and β-phase precipitates along slip lines formed during the HPT deformation (Figure 2d–f). The lower the temperature of preliminary annealing, the more blurry precipitate boundaries could be observed. Heterogeneity of chemical composition and irregular shape of eutectic and the presence of fine discontinuous precipitation may be the reason that the β-phase observed in the initial samples annealed at 400 and 500 °C dissolved easier and had more blurry boundaries after the HPT. Moreover, the primary precipitates and eutectics in the samples annealed at 400 and 500 °C showed slightly lower hardness (Table 3) than precipitates that appeared after annealing at 600 °C, which contained more Ag content (Table 2) and were characterized by a rounded shape and uniform composition.

**Figure 5:**
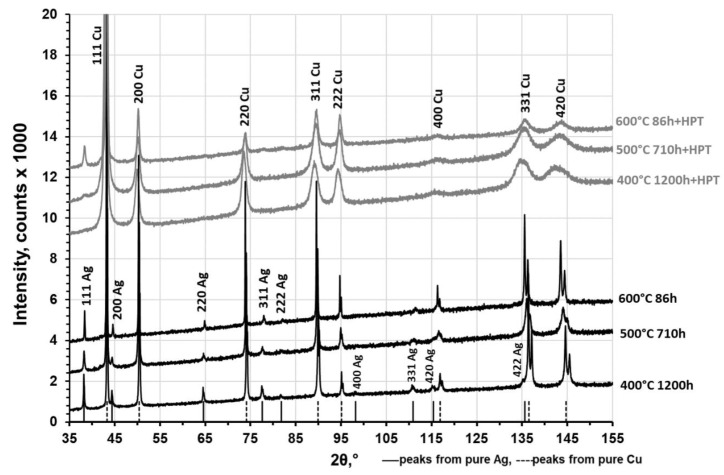
X-ray diffraction patterns of the Cu–8wt.%Ag alloy after annealing at different temperatures before and after HPT.

Figure 5 presents XRD patterns of the Cu–8wt.%Ag alloy after annealing and after the HPT deformation. The Ag and Cu peaks were observed in all the samples after annealing. The Cu peaks (α-phase) observed at higher diffraction angles (137 and 144°) were split, which indicated low internal stresses and larger grain sizes in the annealed samples. The significant broadening of Cu peaks was observed after HPT suggesting a strong grain refinement and significant micro-distortion of the crystal lattice. A slight shift of Cu peaks to lower diffraction angles after HPT was observed as well. This corresponds to the increase of Cu lattice parameters due to migration of Ag atoms from the β-phase (Ag particles) into the Cu-matrix. At the same time the (111) reflection of the β-phase became smaller and more broadened (in the samples annealed at 500 and 600 °C prior to deformation), while the remaining β-phase peaks disappeared or became blurred and undetectable. The disappearance of most β-phase reflections after HPT indicates the partial dissolution of this phase. 

**Figure 6:**
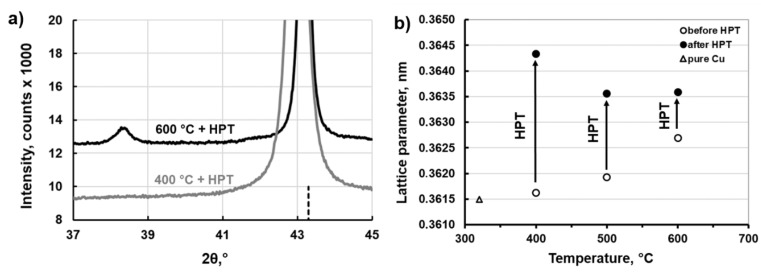
(**a**) Enlarged part of X-ray diffraction patterns of samples of the Cu–8wt.%Ag alloy after HPT and preliminary annealed at 400 and 600 °C. Vertical dotted line shows the (111) peak position of pure copper; (**b**) Changes of lattice parameter of Cu-matrix induced by HPT in samples preliminarily annealed at 400, 500 and 600 °C.

Figure 6a shows an enlarged part of the X-ray diffraction patterns of the deformed samples preliminarily annealed at 400 and 600 °C. It is easy to note that maximum broadening and maximum shift of (111) Cu peak is observed in the sample annealed at 400 °C. Additionally the (111) Ag peak disappeared after HPT in the sample. It confirms that the maximum grain refinement and maximum dissolution of β-phase takes place there.

The changes of lattice parameter of Cu-matrix before and after HPT are graphically presented in Figure 6b. Generally, doping copper with silver leads to an increase in the lattice parameter of copper [23,24]. One can see from Figure 6b that along with the increase of annealing temperature, the lattice parameter increases, because the solubility of Ag in Cu increases alongside the solvus line on the phase Cu–Ag diagram. HPT resulted in the increase of the lattice parameters values, wherein their maximum increase is observed in the sample preliminary annealed at 400 °C. These results show a good correlation with the data presented in Table 1, i.e., the largest amount of dissolved β-phase under action of HPT process is observed in the sample pre-annealed at 400 °C, while the smallest one in the sample pre-annealed at 600 °C. Therefore, it can be concluded that the lower the annealing temperature, the greater the enrichment of matrix in Ag after HPT. It is a result of partial dissolution of the primary β-phase, (α + β) eutectic and probably the effect of complete dissolution of the fine discontinuous precipitates. It should be also noted, that the lattice parameter of Cu-matrix obtained after HPT in the samples pre-annealed at 400, 500 and 600 °C reached 0.36433, 0.36356 and 0.36359 ± 0.00001 nm, respectively. According to the [24] database, a slightly smaller lattice parameter of 0.36421 nm in the sample pre-annealed at 400 °C, corresponds to the Cu–5.82 at.%Ag alloy obtained after homogenizing at 760 °C for 48 days and 780 °C for 70 days. The composition of 5.82 at.%Ag corresponds to 9.5 wt.%Ag, while 8 wt.%Ag is the maximum amount of Ag which can be dissolved in Cu at eutectic temperature 780 °C. In other words, HPT led to a greater dissolution of Ag than the possible maximum solubility of Ag in the Cu–8wt.%Ag alloy obtained after prolonged annealing at 780 °C. This effect of increasing solubility limit of Ag induced by HPT was not observed in the other deformed samples (pre-annealed at 500 and 600 °C) of the examined alloy.

The phenomenon of anomalous phase transitions, i.e., dissolution of the second phase under the influence of SPD, was observed in many works [9,25] and systematically studied in Fe-based alloys [26,27]. For example, it was shown that the C, N and Niwas transferred from the second phase particles into the iron solid solution as a result of SPD, wherein the concentration of these elements could exceed the equilibrium saturation concentrations [26]. The authors presented the following scheme of such a dissolution. At the first stage of the SPD the particles were refined, at a more developed stage of deformation the contribution of “non-crystallographic” deformation mechanisms increased, accompanied by an intense generation of vacancies. Then the separation and drift of atoms from particles in the field of edge dislocations became more efficient. Such phase transitions are also characterized by anomalous rapid diffusion, which can be explained by the generation of excess vacancies and grain boundary diffusion in ultra-fine-grained materials with a large fraction of high angle grain boundaries, and it happened despite the fact that the applied pressure additionally slowed down the diffusion [28,29]. It should be noted that the HPT-induced mass transfer led not only to the increase of the solubility limit, but also to obtaining nanocomposites from mixtures of micro-powders even for immiscible systems [30].

**Figure 7:**
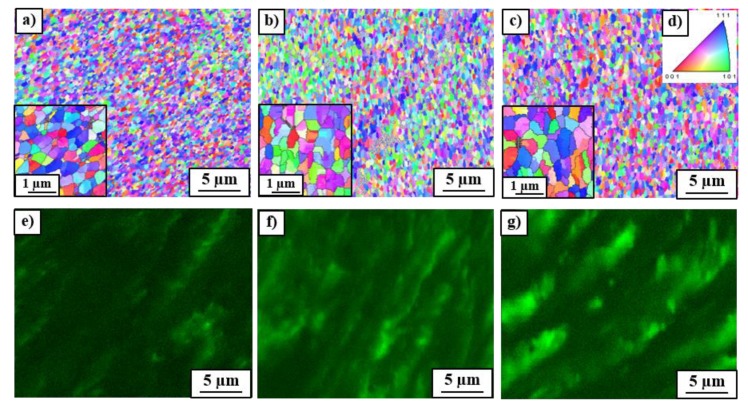
(**a**–**c**) SEM/EBSD orientation maps and (**e**–**g**) corresponding Ag distribution (green color) in Cu-matrix (black color) of the Cu–8wt.%Ag alloy after HPT, preliminary annealed at (**a**,**e**) 400 °C, (**b**,**f**) 500 °C and (**c**,**g**) 600 °C together with (**d**) standard unit triangle for fcc α-phase. The inserts inside (**a**–**c**) display enlarged areas of the EBSD maps with high angle grain boundaries (HAGBs) larger than 10°.

Figure 7 shows SEM/EBSD orientation maps (a–c) and corresponding chemical composition maps (e–g) of the deformed alloy. The character of silver distribution in the form of elongated coarse β-phase particles well correlates with the microstructure observed in Figure 2d–f. The average grain sizes of deformed Cu-matrix (measured on the basis of orientation topography maps) reached 0.35 ± 0.12, 0.44 ± 0.16 and 0.48 ± 0.17 µm, respectively for the samples preliminary annealed at 400, 500 and 600 °C. It seems that the presence of the discontinuous precipitates (with the maximum fraction in the sample annealed at 400 °C) in the form of fine duplex structure additionally leads to the grain refinement of the microstructure during HPT. The statement that the presence of fine dispersed particles promotes the microstructure refinement during SPD has been confirmed in works [7,8]. For example, the nanosize Al_3_(Sc,Zr) precipitates play an important role in the grain refinement of the Al–0.2Sc–0.1Zr alloy subjected to the process of accumulative continuous extrusion forming [8]. The grain size of the alloy dramatically refined from 100 µm to 800 nm through continuous dynamic recrystallization (CDRX). The nanosize particles promoted grain refinement through three mechanisms: the precipitates (i) facilitated retention of high dislocation density in the alloy by enabling the generation of dislocation and pinning dislocation slip, which increased the driving force for CDRX; (ii) promoted the formation of deformation bands, providing sites for activation of CDRX, and (iii) activated CDRX near the grain boundary. The texture of HPT processed samples of the Cu–8wt.%Ag alloy did not show significant differences in intensity and component position. All samples yielded a typical shear texture with A, B, C components of fcc metals. It strongly suggested that the discussed particles did not change the deformation mechanism during the HPT process. 

The results of microhardness measurements are presented in Table 3. The Cu and Ag-solid solutions have the same fcc lattice and the hardness values of Cu-matrix and Ag precipitates are also similar reaching about 134 H_v_ before HPT. After HPT only, the microhardness of the sample pre-annealed at 600 °C was measured, in which the Cu-matrix did not include fine Ag particles resulting from discontinuous precipitation. HPT led to the increase of microhardness of both phases to the value of about 310 H_v_. The increase of microhardness was associated with an increase in the crystal structure defect density and with the increase of high-angle grain boundary fraction as a result of grain refinement induced by HPT. 

Based on results obtained in Ref. [25], the effect of SPD on phase transformations was established to depend strongly on diffusion activation energy and mixing enthalpy of the alloying elements. The higher the activation enthalpy of diffusion, the lower the diffusion relaxation of crystallographic defects formed by SPD. This, in turn, promoted grain refinement and phase transitions (such as dissolution of the second phase or the decomposition of supersaturated solid solution). For example, it was shown that the higher the activation enthalpy of diffusion of the second component (like Ag, Co, Hf, Cr in the Cu-based alloys), the higher the as-called effective temperature (*T*_eff_), which corresponded to a new position of the deformed alloy in the equilibrium phase diagram [25]. In other words, the phases forming during SPD at ambient temperature can also appear after long annealing at a certain elevated temperature *T*_eff_ with subsequent quenching. However, if the real annealing at the elevated temperature led to grain growth, the SPD resulted in the grain refinement of microstructure. 

The effect of SPD on phase transformation of compounds with a positive or negative enthalpy of mixing was different. Due to strong interatomic bonds such as compounds with a negative enthalpy of mixing (such as Cu–Sn, Cu–In), the process of dissolution of second phase practically did not occur [31,32] compared with compounds of positive mixing enthalpy (Cu–Ag, Cu–Ni, Cu–Co). For example, in as-cast Cu–36wt.%Sn alloy which was subjected to HPT at room temperature [31], the microstructure of the alloy before HPT, contained alternating coarse-grained or even single-crystalline plates of the hard intermetallic ζ (Cu_10_Sn_3_) and ε (Cu_3_Sn) phases. After HPT, neither dissolution of phases, nor phase transformations were observed. Only slight grain refinement took place inside the ζ and ε plates, however, the shape of alternating plates remained unchanged.

## 4. Conclusions

The microstructure of the Cu–8wt.%Ag alloy after annealing at 400 and 500 °C contained coarse (α + β) eutectic, coarse β-phase precipitates and fine (α + β) duplex structure formed due to the discontinuous precipitation. The volume fraction of discontinuous precipitates after annealing at 500 °C was smaller than that after annealing at 400 °C. Annealing of the alloy at 600 °C resulted in the transformation of eutectic precipitates into coarse homogeneous β-phase particles. The higher the temperature of annealing, the lower volume fraction of β-phase precipitates and the higher content of Ag in the Cu-matrix (α-phase).

Applying HPT to the Cu–8wt.%Ag alloy resulted in: (1) strong grain refinement of the Cu-matrix (down to about 400 nm); (2) partial dissolution of coarse (α + β) eutectic and coarse β-phase particles, their fragmentation and refinement; (3) dissolution of fine discontinuous precipitates; and (4) an increase of solubility limit of Ag in the Cu-matrix in the sample pre-annealed at 400 °C.

The maximum HPT effect on grain refinement of the Cu-matrix and the dissolution of β-phase was observed in the sample preliminary annealed at 400 °C due to the fact that the initial state of this sample was characterized by: (1) the largest volume fraction of dispersed duplex structure; (2) the presence of a slightly softer (α + β) eutectic in comparison with coarse and homogeneous Ag particles; and (3) the lowest solubility of Ag in Cu-matrix. Specifically, small, heterogeneous precipitates of the irregular shape dissolved more easily than those of large sizes, rounded shape and uniform composition. 

## Figures and Tables

**Table 1:** The volume fraction (%) of β-phase precipitates in the Cu–8wt.%Ag alloy after annealing at different temperatures *T* (°C) measured on the bases of XRD data.

*T* °C	Volume Fraction %		
	Before HPT	After HPT	Increment Δ
400	8.6	2.6	6.0
500	6.6	3.5	3.1
600	6.3	3.9	2.4

**Table 2:** Distribution of silver (wt.%) in the Cu–8wt.%Ag alloy before and after HPT process measured by EDS/SEM.

*T* °C	Before HPT		After HPT	
	Cu-Matrix	Coarse Ag Precipitates	Cu-Matrix	Coarse Ag Precipitates
400	4.0 ± 0.4	32.5 ± 0.6	5.6 ± 0.2	14.1 ± 0.7
500	4.7 ± 0.5	44.1 ± 0.9	5.4 ± 0.2	33.8 ± 0.7
600	5.0 ± 0.4	52.3 ± 1.0	5.1 ± 0.2	39.2 ± 0.8

**Table 3:** Microhardness of Cu-matrix and coarse Ag precipitates in the Cu–8wt.%Ag alloy before and after HPT process.

Treatment	Microhardness H_v_	
	Cu-Matrix	Ag Precipitates
400 °C	132 ± 8	134 ± 6
500 °C	131 ± 5	128 ± 10
600 °C	136 ± 8	142 ± 8
600 °C + HPT	311 ± 20	310 ± 32
